# Neuroimmune pathways involvement in neurodegeneration of R6/2 mouse model of Huntington’s disease

**DOI:** 10.3389/fncel.2024.1360066

**Published:** 2024-02-20

**Authors:** Emanuela Paldino, Giorgia Migliorato, Francesca R. Fusco

**Affiliations:** ^1^Laboratory of Neuroanatomy, Fondazione Santa Lucia IRCCS, Rome, Italy; ^2^Department of Life Sciences, University of Trieste, Trieste, Italy

**Keywords:** pSHP-1, SIRPα, CD47, pSTAT1, microglia, neurodegeneration, Huntington’s disease

## Abstract

Mechanisms of tissue damage in Huntington’s disease (HD) involve excitotoxicity, mitochondrial damage, and neuroinflammation, including microglia activation. CD47 is a membrane protein that interacts with the inhibitory immunoreceptor SIRPα. Engagement of SIRPα by CD47 provides a downregulatory signal that inhibits host cell phagocytosis, promoting a “don’t-eat-me” signal. These proteins are involved in the immune response and are downmodulated in inflammatory diseases. The involvement of inflammation and of the inflammasome in HD has already been described. In this study, we focused on other factors that can be involved in the unregulated inflammatory response that accelerates and exacerbate the neurodegenerative process in HD. Our results show that CD47 on striatal neurons decreased in HD mice, while it increased in wild type mice with age. SIRPα, on the other hand, was present in neurons in the wild type and increases in the R6/2 mice at all stages. Recruitment of SIRPα and binding to CD47 promotes the activation through phosphorylating events of non-receptor protein tyrosine phosphatase SHP-1 and SHP-2 in neurons and microglia. SHP phosphatases are able to curb the activity of NLRP3 inflammasome thereby reducing the detrimental effect of neuroinflammation. Such activity is mediated by the inhibition (dephosphorylation) of the proteins signal transducer and activator of transcription (STAT). We found that activated SHP-1 was present in microglia and neurons of WT mice at 5 and 13 weeks, increasing with time; while in R6/2 it was not localized in neurons but only in microglia, where it decreases with time. Consequently, STAT1 was overexpressed in neurons of R6/2 mice, as an effect of lack of modulation by SHP-1. Thus, our results shed light on the pathophysiology of neuronal damage, on one hand, paving the way toward a modulation of signal transducer proteins by specific inhibitors to achieve neuroprotection in HD, on the other.

## 1 Introduction

Huntington’s disease (HD) is a devastating neurodegenerative disorder that affects millions of people worldwide. This inherited genetic disorder is characterized by the progressive degeneration of nerve cells in the brain, leading to a wide range of motor, cognitive, and psychiatric symptoms. While the genetic underpinnings of HD have been extensively studied, recent research has shed light on the role of neuroinflammation, on one hand, and of phagocytosis, on the other. Phagocytosis is a fundamental cellular process in the pathogenesis of this disease. Indeed, phagocytosis, the process by which cells engulf and digest foreign particles or cellular debris, plays a crucial role in maintaining brain homeostasis. In the context of Huntington’s disease, aberrations in phagocytic activity within the central nervous system are emerging as a critical factor in disease progression. Neuroinflammation, on the other hand, includes several pathways and cytokines, and it is plausible that several of them play an important role in HD pathology (Han et al., 2012). Recently, focus has turned to the NLRP3 inflammasome, that we studied in the R6/2 mouse model of HD, thereby contributing to the understanding of the involvement of inflammatory processes in neurodegenerative disorders (Paldino et al., 2020; Paldino and Fusco, 2022).

Both phagocytosis and neuroinflammation involve the activity of the microglia. Microglia are immune cells that reside in the central nervous system (CNS) where they exert an increased phagocytosis, particularly in periods of neuronal growth and remodeling (Silverman and Wong, 2018; Wilton et al., 2019). The regulation of microglial phagocytosis is important to understand neurodegenerative disorders, where dysregulated microglia phagocytosis is involved in the process of tissue damage and neuronal death (Perry et al., 2010; Sellgren et al., 2019). Microglial cell surface receptors have been identified to regulate phagocytosis over development (Fu et al., 2014) as well as neuron-derived signal receptors. One of cell surface receptor expressed by phagocytic cells, which acts as “don’t eat me” signal to reduce phagocytosis is the signal-regulatory protein alpha (SIRPα) (Elward and Gasque, 2003; Jiang et al., 2020).

In the present study, we directed our interest in exploring several factors involved in phagocytosis such as the expression of SIRPα and its receptor CD47, in the mouse model of HD, to better understand the mechanisms that are at the foundation of neuronal death. The SIRPα-CD47 system activates the non-receptor protein tyrosine phosphatases, namely Src homology region 2 domain-containing phosphatase-1 (SHP-1) and Src homology 2 (SH2) domain-containing tyrosine phosphatase-2 (SHP-2), at the level of cell membrane, in response to extracellular stimuli, exerting its action of inhibiting phagocytosis (Khanna et al., 2007). SHP-1 belongs to the protein tyrosine phosphatase (PTP) family and plays a crucial role in the regulation of signaling pathways involved in immune response and cell growth. Dysregulation of SHP-1 has been implicated in various diseases, including cancers and autoimmune disorders (Chong and Maiese, 2007; Murata et al., 2018). SHP-2 is a non-receptor protein tyrosine phosphatase (PTP) ubiquitously expressed. It has important functions in cell proliferation and differentiation (Agazie et al., 2003) particularly as a response to factors such as epidermal growth factor and platelet-derived growth factor-induced Ras-Raf-Erk cascade (De Rocca Serra-Nédélec et al., 2012). Compelling evidence shows that SHP-2 is also implicated in immune signaling and inflammatory response; indeed, SHP-2 activation prevents overactivation of NLRP3 inflammasome, and therefore, in normal conditions, modulates inflammation and controls tissue damage (Guo et al., 2017).

With respect to the activity of SHP-1 and SHP-2, in this study we have also considered the signal-transducers and activators of transcription (STAT). STATs are important signaling molecules that can activate neuroinflammation. Activation and inactivation of STATs are regulated by phosphorylation and dephosphorylation of tyrosine residues of STATs (Shuai et al., 1993). SHP-1 negatively regulates STAT signaling pathways induced by interferon. It has strong anti-inflammatory roles, as evidenced by the fact that IFN-γ- and LPS-induced iNOS (inducible nitric oxide synthase) expression is higher in SHP-1-deficient macrophages than in wild-type (WT) cells (Hardin and Yu, 2006; Christophi et al., 2009). Thus, we investigated the expression of SHP-1 and SHP-2 and of STAT1/3 to better understand their importance in relation with neuroinflammation in the pathology of HD, in the effort to find a possible therapeutic strategy to fight HD.

## 2 Materials and methods

### 2.1 Animals and tissue processing

All animal experiments, which satisfied ARRIVE guidelines, were performed in accordance with European Communities Council Directive (2010/63 EU) as adopted by the Santa Lucia Foundation Animal Care and Use and approved by Italian Ministry of Health. Transgenic R6/2 mice were obtained by ovarian transplant of hemi zygote females × B6CBAF1/J males, all obtained from Jackson Laboratories (Bar Harbor, ME). F1 mice were used to perform all experiments. Eight mice per group were used. No randomization was performed. The study groups included: R6/2 mice at 5 weeks of age, R6/2 mice at 13 weeks of age, Wild-type mice at 5 and 13 weeks. Mice were handled under the same conditions by one investigator at the same day and time. Genotyping was performed at 21 days of age, and all mice were weaned and housed four in each cage under standard conditions (room temperature: 20 ± 2 °C; humidity: 60%) and a 12/12-h light/dark cycle (7:00 am–7:00 pm) with *ad libitum* access to food and water. Animals (8 R6/2 5 weeks, 8 R6/2 13 weeks, 8 wild type 5 weeks and 8 wild type 13 weeks) were transcardially perfused under deep anesthesia with saline solution containing 0.01 ml heparin, brains were removed and cut in half. One half brain was post-fixed in 14 ml of 4% paraformaldehyde, 10% sucrose and 20% glycerol in 0.1 M phosphate buffer (PB) at + 4 °C for 48 h. Sectioning was performed on a sliding frozen cryostat at 30 μm thickness. Observers who were in blind, collected primary data.

### 2.2 Immunohistochemical studies of mice brain tissue

#### 2.2.1 Evaluation of CD47 neuronal expression

Coronal brain sections were incubated with the marker CD47 (BS-21460R, rabbit polyclonal anti-CD47, Bioss, USA) at 1:200 dilution in a 0.1 M PB solution containing 0.3% Triton X-100 for 72 h at 4°C. Sections were rinsed three times for 5 min at room temperature and subsequently incubated with the secondary biotinylated antibody, 1:200 (biotinylated goat anti-rabbit, Jackson Immunoresearch, West Grove, PA, USA). The immunohistochemical staining was completed with the Streptavidin-Cy3 (Sigma-Aldrich, Merck). All sections were rinsed three times for 5 min at room temperature and subsequently counterstained with Neurotrace™ (Nissl-like fluorescent marker, Jackson, MS, USA) to visualize the number and the immunofluorescence intensity of neurons expressing CD47. Sections were mounted on specimen slides; cover slipped with Anti Fade™ Mounting Medium (Immunological Sciences, Italy) and a confocal laser-scanning microscope (Zeiss LSM 800) was used to acquire all images. The single plane confocal images were acquired under no saturation conditions, with a × 20 objective gaining a × 1 zoom with value 0 of Offset and producing images in the format 1024 × 1024, Airy Units 1.0. A sample of about 100 neurons for each of four sections obtained by the 8 mice per group were analyzed, in order to determine the distribution and the immunoreaction product of CD47 in the striatum of R6/2 and wild-type mice at the different time points 5 and 13 weeks.

#### 2.2.2 Immunohistochemistry for SIRPα

To evaluate the involvement of SIRPα/CD47 signaling pathway, we performed an immunohistochemistry for SIRPα. Peroxidase-antiperoxidase diaminobenzidine tetrahydrochloride single-label immunohistochemistry for Sirpα was performed to identification and distribution of this phagocytosis marker in the mice striatum. Serial sections from rostral neostriatum through the level of anterior commissure (interaural 4.66 mm/Bregma 0.86 mm to interaural 3.34 mm/Bregma-0.46 mm) for three animals per groups, were incubated with mouse anti-SIRPα at 1:200 dilution in 0.1 M PB solution containing 0.3% Triton X-100 for 72 h at 4 °C (NB100-65530 mouse anti-SIRPα, Novus Biologicals, Italy). Subsequently, sections were incubated with mouse peroxidase–antiperoxidase complex diluted 1:100 in 0.1 M PB solution with 0.3% Triton X-100 at room temperature for 1 h. After peroxidase–antiperoxidase incubation, sections were incubated in Tris–HCl buffer containing 10 mg diaminobenzidine tetrahydrochloride for 2 min, adding 15 μl of 3% hydrogen peroxidase. The peroxidase–antiperoxidase diaminobenzidine tetrahydrochloride-labeled sections were then washed in distillated water, placed in 0.1 M PB, mounted on gelatin-coated slides, dried, dehydrated and coverslipped. SIRPα positive cells visualization was performed on collected images with a × 40 and × 100 objective, obtained by Neurolucida™ Stereo Investigator software (Zeiss, Rochester, NY, USA). To analyze the expression of SIRPα in microglia and astrocyte cells, double immunofluorescence were performed with the primary antibodies at 1:500 dilution against IBA-1 (NB100-1028, goat anti-IBA1, Abcam, Novus Biologicals, Italy) and GFAP (mouse anti-GFAP, Immunological Sciences, Italy) in a 0.1 M PB solution containing 0.3% Triton X-100 for 72 h at 4 °C. Microglia and astrocytes in the area of interest were captured performing Z-stacks acquisitions using a × 20 objective increasing the × 1.5 zoom and images in the format 1024 × 1024 were produced.

#### 2.2.3 Analysis of the Src homology 2 domain tyrosine phosphatases SHP-1 and SHP-2 in R6/2 mice brain

Double-label immunofluorescences were carried out to evaluate the distribution of activated SHP-1 and SHP2 in the mice striatum. Coronal brain sections of mice were incubated with specific antibodies for the phosphorylated proteins: SHP1 (ABP11214 rabbit anti phospho SHP1, Immunological Sciences, Italy) or SHP2 (ABP11215 rabbit anti phospho SHP2, Immunological Sciences, Italy) at 1:200 dilution and the primary antibodies against IBA-1 and GFAP protein at 1:500 dilution. Mice brain sections were, also, incubated with the primary antibody pSTAT1 (ABP-0453 rabbit anti pSTAT-1, Immunological Sciences, Italy). After that, sections were rinsed three times for 5 min at room temperature and subsequently incubated with the Alexa Fluor 555 and 488 secondary antibodies (Immunological Sciences, Italy) for 2 h at room temperature at 1:200 dilution in a 0.1 M PB solution containing 0.3% Triton X-100. Sections were then mounted on slides cover slipped with Anti Fade™ Mounting Medium (Immunological Sciences, Italy). A confocal laser scanning microscope (Zeiss LSM 800) was used to acquire all the images. Three separate fields (dorsolateral, central and medial each 1 mm in diameter) in each of three rostro caudally spaced sections of eight mice per group were examined. pSHP1 e pSHP2 confocal images were acquired under no saturation conditions performing Z-stacks acquisitions using a × 20 objective increasing the × 1.0 zoom and images in the format 1024 × 1024 were produced. pSTAT1 single plane images were acquired with a × 20 objective gaining a × 0,8 zoom with value 0 of Offset and producing images in the format 1024 × 1024 and Airy Units 1.0. The same set configuration was performed for all samples.

### 2.3 Western blotting

Dissected striata from the Wt and R6/2 half brains were homogenized with the RIPA lysis buffer containing a protease and phosphatase inhibitor cocktail (Sigma Aldrich, USA) and centrifuged at 13,000 × *g* for 20 min, to obtain a total lysate containing neurons and microglial proteins. Equal amounts of protein were separated using sodium dodecyl sulfate-polyacrylamide gel electrophoresis, transferred to polyvinylidene fluoride membranes, and incubated with CD47 (1:500), SIRPα (1:500), pSHP-1 (1:1000), total STAT-1 (AB-81448 rabbit anti STAT-1, Immunological Sciences, Italy) (1:1000), pSTAT-1 (1:1000), pSTAT3 (ABP-5041 rabbit anti pSTAT3, Immunological Sciences, Italy) (1:1000) and mouse GAPDH-HRP (1:10.000, Immunological Sciences, Italy) antibodies, overnight at 4 °C. After being washed with Tris-buffered saline (TBS)/Tween 20, membranes were incubated with HRP-labeled secondary antibodies. Proteins signal was visualized using the Invitrogen iBright CL 1500 Imaging system.

### 2.4 Statistical analysis

All the collected images have been quantified by using the Java image processing and analysis program Fiji ImageJ. Cells of interest were selected using the freehand tool. From the Analyze menu, Set measurements Mean “Gray Value,” “Area,” and “Min and Max Gray Value” were selected. The region characterized by absence of fluorescence was considered in the background and it was subtracted. Finally, the mean values with SEM were obtained for all measures. ANOVA analysis available in the software GraphPad Prism version 8.0 was performed. *P*-values < 0.05 were considered statistically significant.

## 3 Results

In order to evaluate the possible dysfunction of the phagocytosis mechanism, the expression of the main players of this pathway was evaluated in two age stages of R6/2 mice: pre-symptomatic (corresponding to 5 weeks) and symptomatic represented by 13 weeks of age.

### 3.1 Modulation of CD47 protein expression levels in R6/2 mice striatum

**FIGURE 1:**
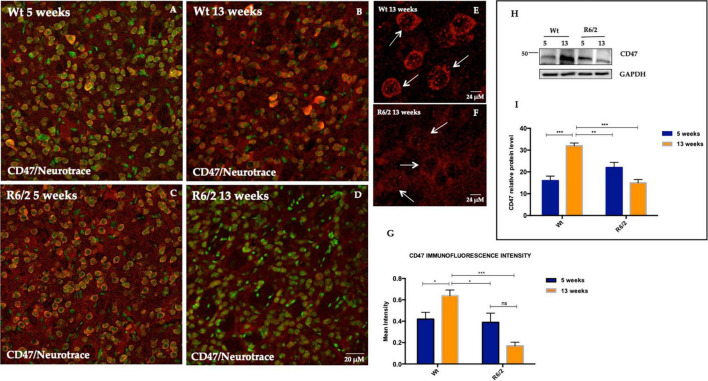
CD47 expression in striatal neurons of R6/2 mice. Confocal laser scanning microscopy images of double-label immunofluorescence for CD47 (visualized in red fluorescence) and the Nissl-like fluorescent marker Neurotrace (visualized in green fluorescence). **(A–D)** Images show the expression pattern of CD47 in the striatum of 5 and 13 weeks-old wild-type mice and in 5 and 13 weeks-old R6/2 mice. **(E,F)** Detail of cytoplasmic membrane CD47 expression in 13 weeks-old Wt mice. In 13 weeks-old R6/2 mice CD47 is lower due to cell death process **(G)** Histogram shows CD47 intensity quantification which is significantly decreased in the striatal neurons of 13 weeks-old R6/2 mice [Genotype effect *F*_(1,153)_ = 11.97, ***P = 0.0007]. **(H,I)** Tukey’s multiple comparisons analysis revealed a significant protein decrease of striatal neuronal CD47, as evaluated by western blotting in R6/2 mice [Genotype effect *F*_(1,20)_ = 8.667, **P = 0.0080; Time effect *F*_(1,20)_ = 5.802, **P = 0.0258; Genotype X Time Interaction *F*_(1,20)_ = 37.89, ***P < 0,0001].

CD47 is a transmembrane protein widely expressed on most mammalian cells, including neurons. The function of this protein in regulating phagocytosis has been established, more specifically by inhibiting it through the recruitment and bonding to SIRPα, expressed on phagocytes (Brown and Frazier, 2001; Gardai et al., 2005). We investigated the expression of CD47 in the mice striatum of pre and fully symptomatic R6/2 compared to their Wt littermates. CD47 was preferentially expressed in the striatal neurons as evidenced by immunofluorescence staining performed on brain tissue of R6/2 and Wt mice at 5 and 13 weeks of age (Figures 1A–D). In the 13 weeks old Wt mice the CD47 *puncta* expression and cytoplasmic membrane distribution appeared more intense compared to 13 weeks old R6/2 mice in which CD47 expression was significantly decreased and its striatal neuron distribution appeared not sharp (Figures 1E, F). The immunofluorescence intensity of CD47-positive striatal neurons was higher in 13 weeks old Wt mice *p* < 0.0001, while a statistically significant reduction of CD47-positive neurons was observed in R6/2 mice at 13 weeks (Figure 1G). Data was confirmed with molecular experiments (Figures 1H, I).

### 3.2 Neuronal signal-regulatory protein alpha (SIRPα) upregulation during neurodegeneration of the R6/2 mice striatum

**FIGURE 2:**
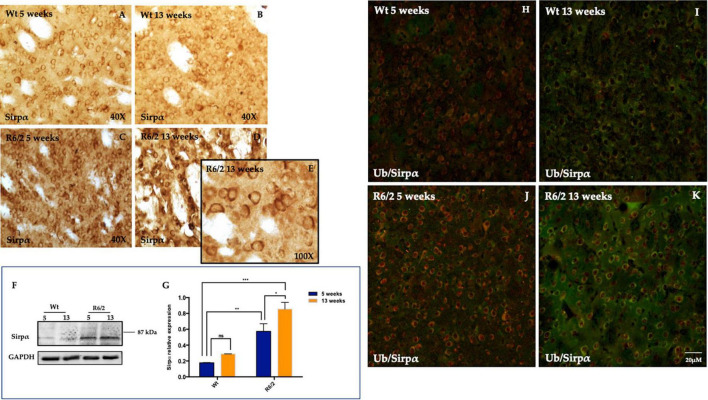
Signal-regulatory protein alpha (SIRPα) distribution in striatal neurons. **(A–D)** Representative transmitted light microscope images showing Dab staining for SIRPα counterstained with Hematoxylin in the striatum of 5 and 13 weeks-old experimental animal groups. **(E)** Higher magnification detail of presynaptic SIRPα expression in 13 weeks-old R6/2 mice. **(F,G)** Representative image of Western Blot and densitometry analysis of SIRPα. Tukey’s multiple comparisons analysis performed on probed membranes revealed a significant increase of the striatal SIRPα protein expression in the 5 and 13 weeks-old R6/2 mice [Genotype effect *F*_(1,16)_ = 56.23, ***P < 0.0001; Time effect *F*_(1,16)_ = 9.423, **P = 0.0073] compared to 5 and 13 week-old Wt. **(H–K)** Representative confocal microscopy images showed the ubiquitin (visualized in red fluorescence), which did not colocalize with SIRPα protein (visualized in green fluorescence).

We performed immunohistochemistry experiments to evaluate SIRPα protein expression in the R6/2 mice striatum and to investigate its expression pattern. Surprisingly, DAB (diaminobenzidine tetrahydrochloride) immunochemistry highlighted a basal neuronal levels of SIRPα protein in Wt animals (Figures 2A, B) and a significant and persistent SIRPα expression in the striatal neurons of R6/2 mice at 13 weeks of age (Figures 2C, D). The characteristic membrane cytoplasmic distribution is referred to presynaptic SIRPα localization which promotes binding with postsynaptic CD47 (Jiang et al., 2022; Figure 2E), however, we cannot rule out the possibility of a postsynaptic localization SIRPα (Ding et al., 2021) (Supplementary Figure 1). DAB immunohistochemistry data were confirmed by molecular experiments performed on striata half-brain lysates derived from the same animals groups on which immunohistochemistry results were obtained (Figure 2F). Since SIRPα was highly expressed in 5 weeks old R6/2 mice striatal neurons and its persisting expression was observed in the surviving neurons of 13 weeks old R6/2 (Figure 2G), we evaluated the possibility that SIRPα protein may be sequestered by the neuronal nuclear inclusions (NIIs), a hallmark of HD. As the antibody against ubiquitin labels NIIs in R6/2 mice, we examined whether SIRPα was ubiquitin immunoreactive in each group. Ubiquitin immunolabeling of striata from wild-type animals did not show any immunoreactive inclusions (Figures 2H, I) but rather a very light diffuse immunolabeling of cell bodies and neuropil. By contrast, and as expected from previous studies in other cell lines and tissues (Giampà et al., 2006), striatal brain sections isolated from 5/13-week-old R6/2 transgenic mice showed the NIIs marked by ubiquitin which did not co localize with SIRPα protein (Figures 2J, K). This ruled out the possibility that SIRPα could be sequestered in NIIs.

**FIGURE 3:**
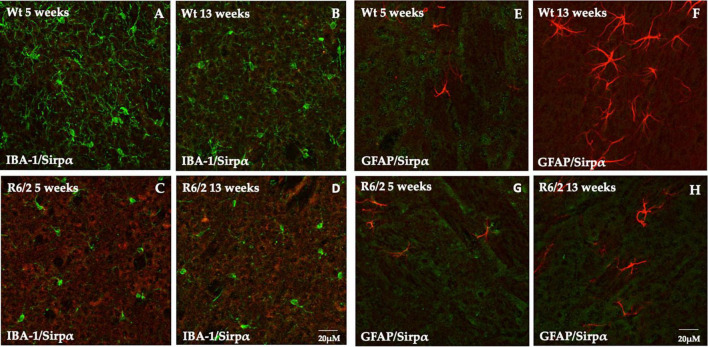
Study of SIRPα expression in microglia and astrocytes cells. **(A–D)** Z-stack confocal laser scanning microscopy images of double-label immunofluorescence for SIRPα (visualized in red fluorescence) and IBA-1 (visualized in green fluorescence). Representative images of mice striatum from all experimental groups show the presence of phenotypically activated microglia in 5 and 13 weeks old R6/2 mice. In all animal groups the colocalization of SIRPα and IBA-1 was undetectable. **(E–H)** Z-stack confocal laser scanning microscopy images of double-label immunofluorescence for SIRPα (in green) and GFAP (in red) of dorsal mice striatum where a higher number of astrocyte is generally observed. In all experimental animals, there was no evidence of colocalization of SIRPα with GFAP positive cells.

Signal-regulatory protein alpha was not detectable in microglia (Figures 3A–D) and GFAP positive cells (astrocytes) were completely devoid of it (Figures 3E–H).

### 3.3 The PTPs activation pattern

In the PTP family, a subgroup of cytoplasmic PTPs characterized by containing two Src homology 2 (SH2) NH2-terminal domains and a C-terminal protein-tyrosine phosphatase domain is referred to as SHP.

**FIGURE 4:**
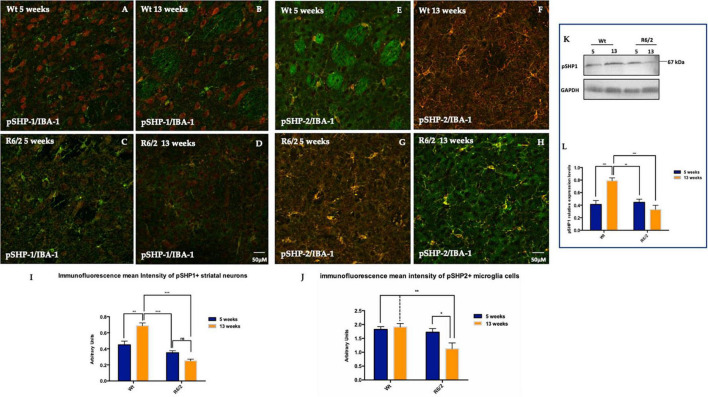
Distribution pattern of phosphorylated SHP-1 and SHP-2. **(A–D)** pSHP-1 is showed in red fluorescence, IBA-1 in green. Microglia expression of pSHP-2 in the R6/2 mice striatum. **(E–H)** pSHP-2 (visualized in red fluorescence) and IBA-1 (visualized in green fluorescence). **(I)** Two-way ANOVA analysis performed on data obtained by 5 and 13 weeks-old mice showed a statistically significant genotype effect of pSHP-1 positive striatal neurons [*F*_(1,44)_ = 65.85, ***P < 0.0001; Time *F*_(1,44)_ = 4.374, **P = 0.00423; Genotype X Interaction *F*_(1,44)_ = 26.35, ***P < 0.0001]. **(J)** Tukey’s multiple comparisons test showed a statistically significant Genotype Effect on microglial pSHP-2 reduction expression in the R6/2 mice at 13 weeks-old of age [*F*_(1,36)_ = 9.094, **P = 0.0047]. **(K,L)** Molecular data analysis of pSHP-1 revealed a significantly decreased in the striatal neurons of 5 and 13 week-old R6/2 mice with a genotype effect [*F*_(1,28)_ = 13.74, ***P = 0.0009; Time *F*_(1,28)_ = 5.283, **P = 0.00292; Genotype X Time Interaction *F*_(1,28)_ = 18.57, ***P = 0.0002].

We investigated the expression pattern of phosphorylated SHP-1 and SHP-2 in the mouse brain of R6/2 mice. R6/2 mice showed a lower expression of neuronal phosphorylated SHP-1 protein from pre-symptomatic to late stages compared to WT control group (Figures 4A–D), in which pSHP-1 protein expression increased during the time (Figure 4I). The presence of pSHP-1 was, also, observed in cells whose morphology can be traced back to the microglial one (Supplementary Figure 2). The immunoreaction product of pSHP-2 was not detected in the striatal neurons neither in R6/2 nor in the wild type mice. However, its expression was specifically localized on microglial cells as represented in Figures 4E–H. The immunofluorescence of phosphorylated SHP-2 intensity analysis performed on microglia cells was statistically significant lower in the R6/2 mice at 13 weeks of age (Figure 4J).

**FIGURE 5:**
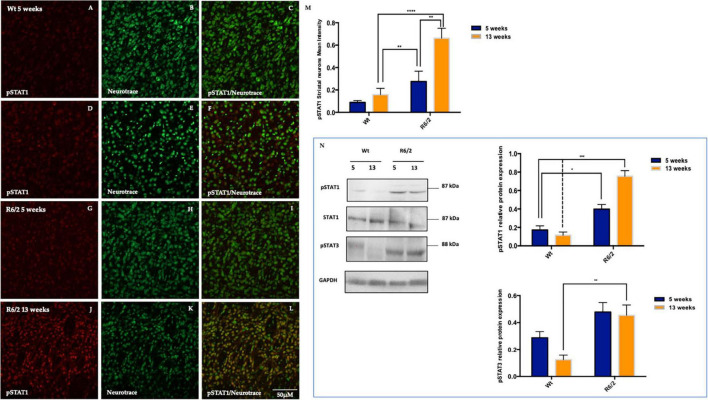
Representative confocal microscopy images of pSTAT1 immunostaining in R6/2 mice striatum. **(A–L)** Phosphorylated STAT1 is showed in red fluorescence, counterstained with Nissl-like fluorescent marker Neurotrace visualized in green. **(M)** Two-way ANOVA analysis performed on 5 and 13 weeks-old animals group showed a statistically significant effect of genotype of pSTAT1 positive striatal neurons [*F*_(1,32)_ = 24.26, ***P < 0.0001, Time *F*_(1,32)_ = 10.58, **P = 0.0027 and Genotype X Time Interaction *F*_(1,36)_ = 5.102, *P = 0.0309]. Protein quantification analysis of pSTAT1 revealed a significantly increment in the striatal neurons of 5 and 13 week-old R6/2 mice with genotype effect [*F*_(1,12)_ = 80.35 ***P < 0.0001; Time *F*_(1,12)_ = 9.655; **P = 0.0091, Genotype X Time Interaction *F*_(1,12)_ = 18.27; **P = 0.0011]. **(N)** Moreover, molecular experiments data analysis revealed a statistical significant high protein expression of pSTAT3 in the R6/2 mice with a genotype effect [*F*_(1,12)_ = 19.26; **P = 0.009; Time *F*_(1,12)_ = 2.537; *P = 0.1372, Genotype X Time Interaction *F*_(1,12)_ = 1.378; *P = 0.2632].

Since pSHP1 plays an anti-inflammatory role by dephosphorylating pro inflammatory transcription factors role, in this study we evaluated STAT1 activation in the R6/2 mice striatum. In the animals control the expression levels of activated STAT1 are mostly undetectable (Figures 5A–F). Fluorescence images revealed a significant activation of STAT1 expression in 5 weeks-old R6/2 mice that statistically increased in 13 weeks-old R6/2 mice (Figures 5G–M). Molecular biology experiments confirmed what we observed on mice brain tissue, showing also the activation of STAT3 exclusively in the R6/2 mice (Figure 5N).

## 4 Discussion

The injured CNS (e.g., stroke, HD, Alzheimer’s disease, HD) requires an innate immune response in order to curb the damage and trigger the repair of tissue by clearance of apoptotic cells and other toxic debris (Gasque et al., 2000; Barnum, 2002; Nguyen et al., 2002).

To this end, it is crucial that both glia and neuronal cells can recognize non-self, toxic materials, and self, so that they are able to remove apoptotic bodies and further restrict inflammation. In some occasions glia will respond with massive recruitment of peripheral macrophages and reactive microglia, which can represent a threat for CNS (Wyss-Coray and Mucke, 2002). However, neuronal cells can express specific “don’t eat me” signals to diminish an adverse phagocytosis and to induce a restorative inflammatory response by anti-inflammatory cytokines and growth factors that are involved in tissue repair.

The importance of microglia in the health of central nervous system and its involvement in its disease has been the object of many studies. Microglial activation is protective for the injured tissue because of its ability to remove cell debris and to reconstruct the integrity of damaged tissue (Hanisch and Kettenmann, 2007; Lalancette-Hébert et al., 2007; Perry et al., 2010). However, the excessive microglial activation could lead to CNS tissue damage and interfere with CNS repairing mechanisms by releasing toxic substances, such as reactive oxygen species (ROS), pro-inflammatory cytokines (Thored et al., 2009) and nitric oxide (NO).

Several receptors have been described on the surface of microglia to detect the “turning on” and “turning off” signals (“eat me/don’t eat me”) released from other cells in the CNS and play critical role in modulating microglial responses in many CNS injuries and neurodegenerative diseases (Hu et al., 2014). One of these signaling proteins, namely, SIRPα and its ligand CD47, are expressed on the surface of microglia as well as on other types of CNS cells (Gaikwad et al., 2009; Hu et al., 2015). Several studies have documented the importance of the interaction between SIRP and CD47 in mediating the intercellular communication in the CNS. In particular, SIRPα activation has been related to the inhibition of several cell functions, such as cytokine production (Latour et al., 2001; Kong et al., 2007; Gitik et al., 2011), monocyte adhesion to the extracellular matrix, and phagocytosis (Oldenborg et al., 2000; Smith et al., 2003; Liu et al., 2005).

On the other hand, SIRPα has been described on neuronal surfaces, where it seems to exert quite a different function than that on microglia. Indeed, while SIRPα on microglia displays an inhibitory effect on phagocytosis, neuronal SIRPα allows microglial phagocytosis and the fine-tuning of synapses (Ide et al., 2007; Janssen et al., 2008). In our study, we have investigated the expression of signals of phagocytosis in the R6/2 mouse model of HD. We found changes in the SIRPα/CD47 signaling system throughout the course of the disease. Indeed, in the R6/2 mouse we found the expression of SIRPα predominantly on neurons of the striatum, rather than on microglial cells. This expression was constantly higher compared to that of wild type animals at all stages. The relative lower levels of SIRP observed in Wt mice, may suggest that animals are not at the peak of synaptic pruning during which increased microglia activation is observed. This finding is consistent with the effects of neuronal SIRPα mentioned above. In fact, the activity of SIRPα is expected to be higher in case of a disease where an active process of neuroinflammation and tissue damage evokes higher level of phagocytosis.

The immunoreaction of CD47, known to be localized post-synaptically on neurons (Jiang et al., 2022), became less intense in the R6/2 mouse brain, as to indicate that this signaling factor has a somewhat independent function from SIRPα. This is not so surprising if one considers that the mechanism by which CD47 would inhibit phagocytosis depends on the presence of SIRPα on microglial cells. We found, instead, that SIRPα is localized on neurons and exerts its functions as a promoter of phagocytosis. This is also consistent with the findings of Jiang and co-workers that observed that a reduction of CD47 caused and increased microglial phagocytosis, while its overexpression decreased it.

Our study also investigated SHP-1 and SHP- 2 proteins. The Protein tyrosine phosphatase (PTP) family, a subgroup of cytoplasmic PTPs characterized by containing two Src homology 2 (SH2) NH2-terminal domains and a C-terminal protein-tyrosine phosphatase domain is referred to as SHP. They are involved in several cellular activities, such as cytoskeletal maintenance, cell division, and cell differentiation (Bialy and Waldmann, 2005). Of these, two particular SHP proteins known as SHP-1 (SH-PTP1, PTP1C, HCP, and SHP) and SHP-2 (SH-PTP2, PTP1D, Syp, PTP2C, and SH-PTP3) have been linked to trophic factor signaling (You and Zhao, 1997), cell growth, mitogen-activated protein kinase (MAPK) activity and chemotactic responses. SHP-1 was believed to play a negative role in modulating cell function, whereas SHP-2 was believed to up-regulate a variety of cell signal transduction processes. However, given the complex functions performed by SHP-1 and SHP-2, their role has not been completely defined, especially in the field of neurodegeneration. The function of SHP-1 and SHP-2 during cell injury and inflammation are not easily defined, since these PTPs appear to possess dual roles to either enhance or inhibit cell survival under a variety of environmental conditions.

Here, we showed that SHP-1 in its phosphorylated from was expressed both in neurons and the microglia of wild type mice. On the contrary, SHP-1 was only expressed in microglia with no signal of immunoreaction in the neurons of R6/2 mice, as an effect of genotype, even in the early stages of the disease. Such activity is mediated by the activation (phosphorylation) of the protein signal transducer and activator of transcription (STAT1). STATs are important signaling molecules that can activate brain inflammation. Activation and inactivation of STATs are both regulated by phosphorylation and dephosphorylation of tyrosine residues of STATs (You and Zhao, 1997). SHP-1 inhibits STAT signaling pathways induced by interferon.

In our study, in fact, we found that pSTAT1 was overexpressed in the neurons of R6/2 mice brain, as an effect of the lack of inhibition exerted by SHP-1 of which R6/2 mice neurons were devoid. Interestingly, STAT1 inhibition is able to reduce apoptotic cell death (Wu et al., 2016). On the contrary, SHP-2 intensity of immunoreaction decreased with disease progression in R6/2. Interestingly, the activation of SHP-2 is known to modulate inflammation. In fact, Guo and coworkers showed that NLRP3 activation causes SHP-2 to migrate to the mitochondria and prevent the release of proinflammatory mitochondrial DNA and Reactive oxygen species. Thus, such increase in SHP-2 could be interpreted as an attempt to counterbalance the ongoing inflammatory reaction occurring in HD.

Taken together, our data emphasize the importance of phagocytosis and inflammation signals in HD pathology, indicating the possibilities of its modulation as a therapeutic strategy for such a devastating disease. In a recent study, Morozko et al. (2021) showed that knocking down the gene for protein inhibitor of activated STAT1 in HD mice had a normalizing effect on HD transcriptional dysregulation. Thus, our results pave the way toward a modulation of STAT-1 through specific inhibitors that are expected to exert a neuroprotection in HD.

## Data availability statement

The original contributions presented in the study are included in the article/Supplementary material, further inquiries can be directed to the corresponding author.

## Ethics statement

The animal study was approved by Dr. Vincenzo Ugo SANTUCCI, Italian Health Minestery. The study was conducted in accordance with the local legislation and institutional requirements.

## Author contributions

EP: Conceptualization, Data curation, Formal Analysis, Investigation, Methodology, Software, Resources, Supervision, Writing – original draft. GM: Data curation, Resources, Writing – original draft. FF: Data curation, Funding acquisition, Project administration, Supervision, Validation, Visualization, Writing – original draft, Writing – review and editing.

## Supplementary material

Supplementary Figure 1 **(A–C)** Confocal laser scanning microscopy of Z-stack images of double-label immunofluorescence for SIRPα (visualized in red fluorescence) and Synaptophysin (visualized in green fluorescence). Representative images of mice striatum from Wt mice show the colocalization of SIRPα with a presynaptic marker, Synaptophysin. **(D,E)** Confocal laser scanning microscopy of Z-stacks images of double-label immunofluorescence for CD47 (in red) and PSD95 (in green) of Wt mice striatum show the colocalization of CD47 with a postsynaptic marker, PSD95.

Supplementary Figure 2 **(A–H)** pSHP-1 is showed in red fluorescence, IBA-1 in green. Representative confocal laser scanning microscopy images show phosphorylated SHP1 in the striatal neurons of WT animals at 5 and 13 weeks. Furthermore, it is possible to observe pSHP1 immunofluorescence staining in cells whose morphology can be traced back to microglia as highlighted by the arrows.
