# Supplementary figures and images for: Neuroimmune pathways involvement in neurodegeneration of R6/2 mouse model of Huntington’s disease
Source: Front Cell Neurosci. 2024 Feb 20;18:1360066. doi: 10.3389/fncel.2024.1360066 (PMC10912295; doi:10.3389/fncel.2024.1360066)

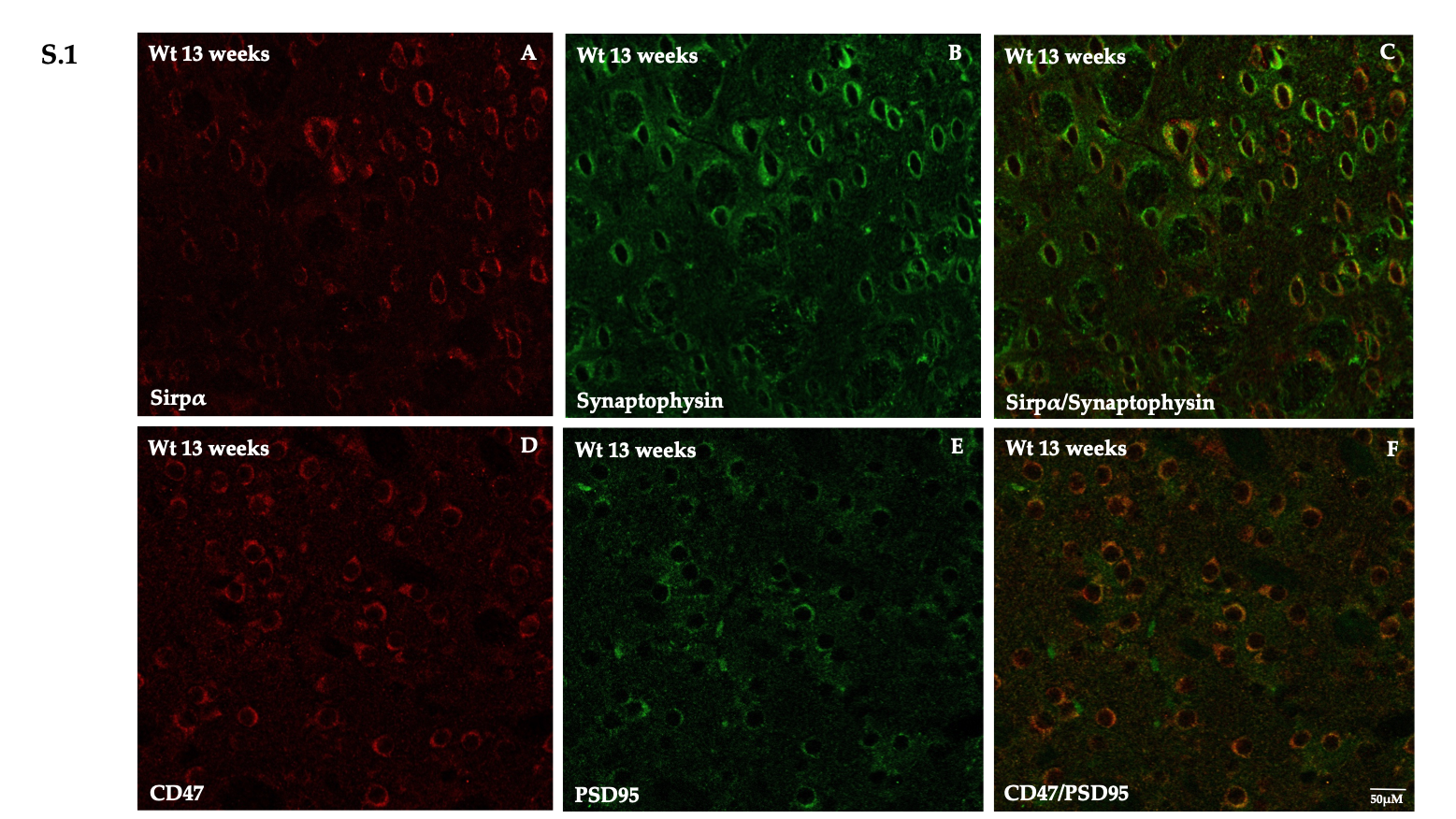

Supplement: Supplementary Figure 1 — (A–C) Confocal laser scanning microscopy of Z-stack images of double-label immunofluorescence for SIRPα (visualized in red fluorescence) and Synaptophysin (visualized in green fluorescence). Representative images of mice striatum from Wt mice show the colocalization of SIRPα with a presynaptic marker, Synaptophysin. (D,E) Confocal laser scanning microscopy of Z-stacks images of double-label immunofluorescence for CD47 (in red) and PSD95 (in green) of Wt mice striatum show the colocalization of CD47 with a postsynaptic marker, PSD95. [file Image_1.tif]

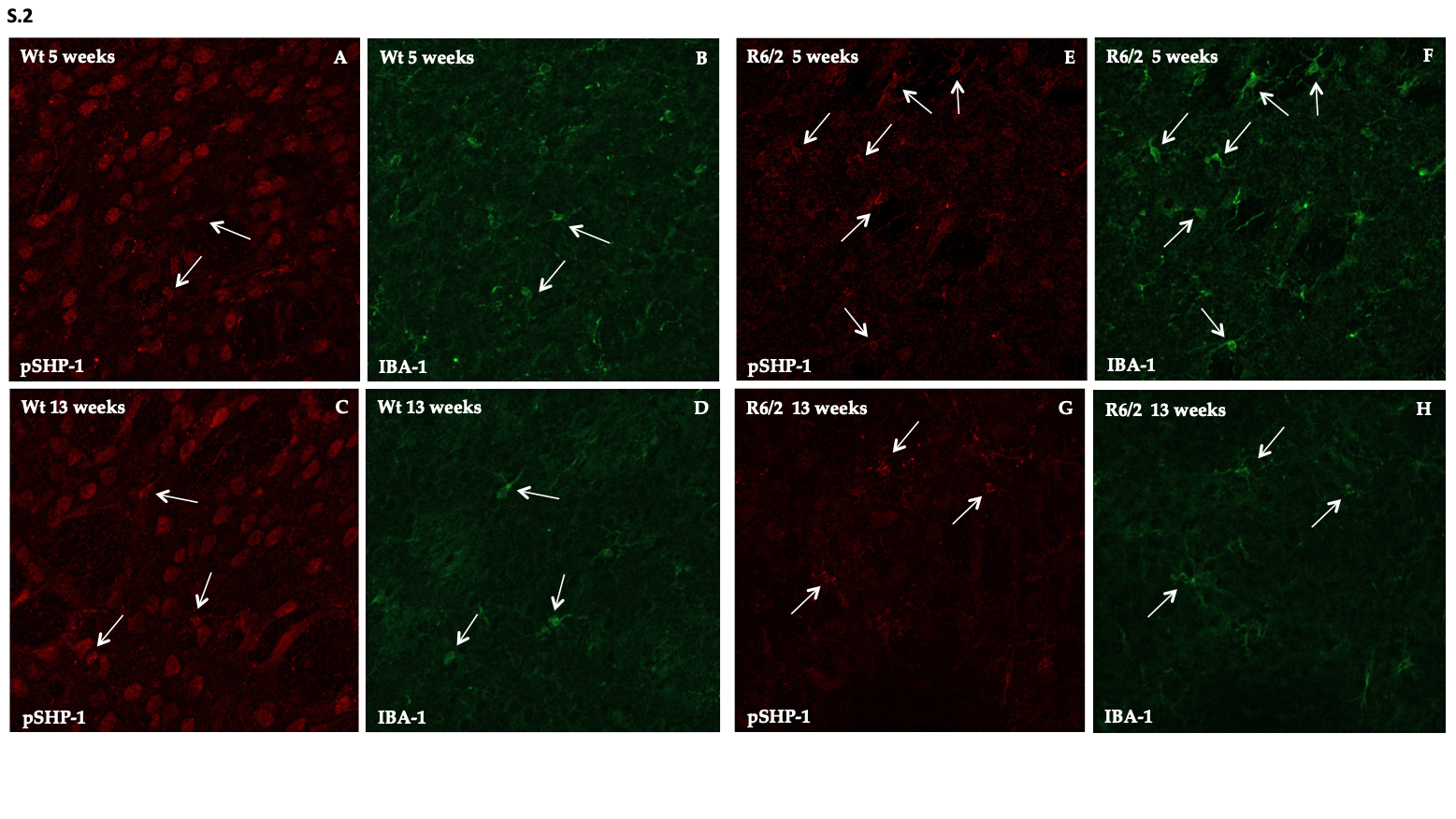

Supplement: Supplementary Figure 2 — (A–H) pSHP-1 is showed in red fluorescence, IBA-1 in green. Representative confocal laser scanning microscopy images show phosphorylated SHP1 in the striatal neurons of WT animals at 5 and 13 weeks. Furthermore, it is possible to observe pSHP1 immunofluorescence staining in cells whose morphology can be traced back to microglia as highlighted by the arrows. [file Image_2.tif]
